# SpoT-Mediated NapA Upregulation Promotes Oxidative Stress-Induced Helicobacter pylori Biofilm Formation and Confers Multidrug Resistance

**DOI:** 10.1128/AAC.00152-21

**Published:** 2021-04-19

**Authors:** Yican Zhao, Yuying Cai, Zhenghong Chen, Huanjie Li, Zhengzheng Xu, Wenjuan Li, Jihui Jia, Yundong Sun

**Affiliations:** a Key Laboratory for Experimental Teratology of the Ministry of Education and Department of Microbiology, School of Basic Medical Science, Cheeloo College of Medicine, Shandong University, Jinan, Shandong, China; b Department of Microbiology, Key Laboratory of Medical Microbiology and Parasitology, Guizhou Medical University, Guiyang, China

**Keywords:** *Helicobacter pylori*, biofilms, antibiotic resistance, SpoT, NapA

## Abstract

Recently, the incidence of drug-resistant Helicobacter pylori infection has increased. Biofilm formation confers multidrug resistance on bacteria. Moreover, it has been found that the formation of biofilms on the surfaces of gastric mucosae is an important reason for the difficulty of eradication of H. pylori. The mechanisms underlying H. pylori biofilm formation *in vivo* have not been elucidated. Reactive oxygen species (ROS) released by the host immune cells in response to H. pylori infection cannot effectively clear the pathogen. Moreover, the extracellular matrix of the biofilm protects the bacteria against ROS-mediated toxicity. This study hypothesized that ROS can promote H. pylori biofilm formation, and treatment with low concentrations of hydrogen peroxide (H_2_O_2_) promoted this process *in vitro*. Comparative transcriptome analysis of planktonic and biofilm-forming cells revealed that the expression of SpoT, a (p)ppGpp (guanosine 3′-diphosphate 5′-triphosphate and guanosine 3′,5′-bispyrophosphate) synthetase/hydrolase, is upregulated in H_2_O_2_-induced biofilms and that knockout of *spoT* inhibited H. pylori biofilm formation. Additionally, this study used weighted gene coexpression network analysis to examine the key target molecules involved in SpoT regulation. The analysis revealed that neutrophil-activating protein (NapA; HP0243) promoted H_2_O_2_-induced biofilm formation and conferred multidrug resistance. Furthermore, vitamin C exhibited anti-H. pylori biofilm activity and downregulated the expression of *napA in vitro*. These findings provide novel insights into the clearance of H. pylori biofilms.

## Abstract (precis)

Recently, the incidence of drug-resistant Helicobacter pylori infection has increased. Biofilm formation confers multidrug resistance on bacteria.

## INTRODUCTION

Globally, the average infection rate of Helicobacter pylori, a common pathogen, is approximately 50%. In some areas of developing countries, the infection rate is as high as 90% (1). Previous studies have demonstrated that H. pylori infection is associated with chronic gastritis, peptic ulcer, and gastric cancer (2). Thus, the alleviation of H. pylori infection can aid in decreasing the incidence of gastric cancer (3).

However, the major challenge for the alleviation of H. pylori infection is the development of drug resistance in the pathogen (4). Biofilm formation confers drug resistance on H. pylori (5–7), especially against common clinical antibiotics. For example, the MICs of clarithromycin, amoxicillin, and metronidazole against H. pylori biofilms were 40-, 40-, and 10-fold higher, respectively, than those against planktonic bacteria (8).

In 2006, Carron and colleagues first reported that H. pylori could form biofilms on the gastric mucosal surface, as determined by using scanning electron microscopy (SEM) (9). The rate of H. pylori biofilm formation on the gastric mucosae of patients with peptic ulcers was 97.3% (10). Cammarota et al. reported that the efficacy of antibiotics against H. pylori biofilms on the gastric mucosae was at least 4 times lower than that against planktonic cells in all patients (11).

Recent studies have demonstrated that H. pylori forms biofilms on the surfaces and in the compartments of the human gastric glands (12). However, the mechanism underlying H. pylori biofilm formation in the stomach has not been elucidated. The elucidation of mechanisms underlying biofilm formation can aid in developing therapeutic strategies to inhibit H. pylori biofilm formation *in vivo* and treat refractory H. pylori infections.

Host immune cells secrete reactive oxygen species (ROS) as the first line of defense against pathogens (13). H. pylori infection elicits a strong inflammatory response upon colonization of the host gastric mucosa. The host inflammatory response is mediated mainly by neutrophils and macrophages, which release ROS and reactive nitrogen species to eliminate H. pylori (14–17).

However, H. pylori can permanently colonize the gastric mucosa, a process mediated through various oxidoreductase systems (18). Biofilms protect the bacteria against the toxic effects of ROS produced by the host immune cells (19). The extracellular matrix of H. pylori is a physical barrier that prevents the diffusion of ROS (20).

Meta-analysis of clinical treatment data has revealed that combination treatment with antioxidants and antibiotics increased the clearance rate of H. pylori (21). This may be attributed to the effects of antioxidants on biofilms. For example, studies have found that vitamin C (Vc) can destabilize bacterial biofilms (22).

These findings suggested that ROS released from the inflammatory cells promote the formation of H. pylori biofilms in the stomach. In this study, the formation of H. pylori biofilms was induced *in vitro* using a low concentration of hydrogen peroxide (H_2_O_2_). Additionally, the mechanism underlying H_2_O_2_-induced biofilm formation was examined using transcriptome sequencing.

## RESULTS

### Oxidative stress promotes H. pylori biofilm formation.

**FIG 1:**
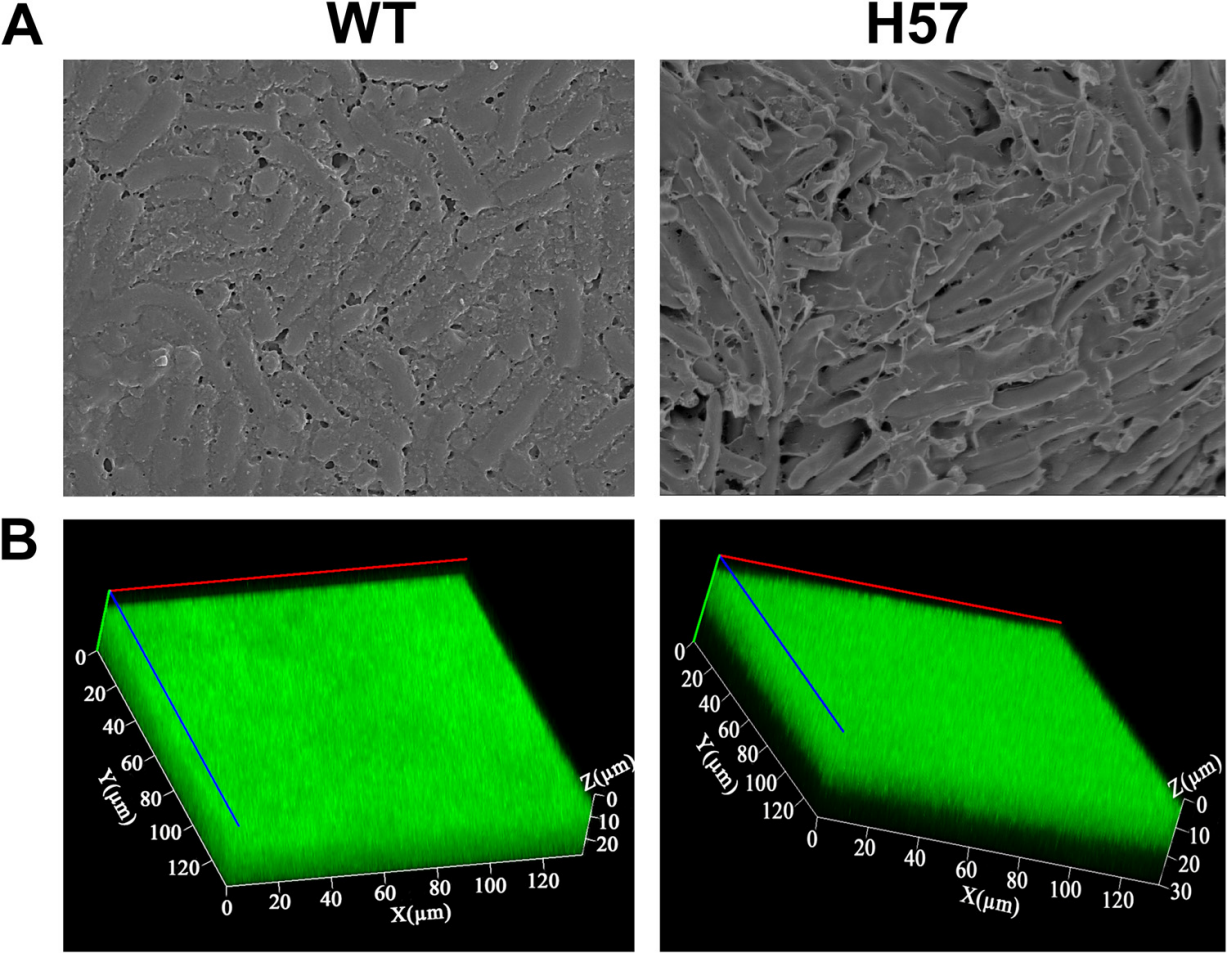
A low concentration of H_2_O_2_ (50 μM) promotes biofilm formation in the wild-type and H57 Helicobacter pylori strains. (A) Confocal laser scanning microscopy images of the biofilm. Cells stained with membrane-permeant SYTO 9 (green) and membrane-impermeant propidium iodide (red) were visualized using confocal microscopy. (B) Scanning electron microscopy images of the biofilm. The biofilm used in this experiment is a mature biofilm grown on a nitrocellulose membrane for 3 days. The planktonic bacteria were from the early-exponential phase (OD_600_, 0.4 to 0.5).

Based on previous studies examining the effects of ROS on bacteria (23, 24), an H. pylori culture was supplemented with low concentrations of H_2_O_2_ to simulate the oxygen stress environment encountered by H. pylori in the human body. The effect of oxidative stress on the induction of H. pylori biofilm formation was examined. Supplementation with 50 μM H_2_O_2_ promoted biofilm formation in H. pylori strain 26695 (the wild-type [WT] strain) and the clinical isolate strain (strain H57) (Fig. 1).

We also found that biofilms induced by H_2_O_2_ were more tolerant to antibiotics than those we had previously induced through nutrient deficiency (see Table S1 in the supplemental material).

### Analysis of differentially expressed genes involved in H. pylori biofilm formation using transcriptome sequencing.

**FIG 2:**
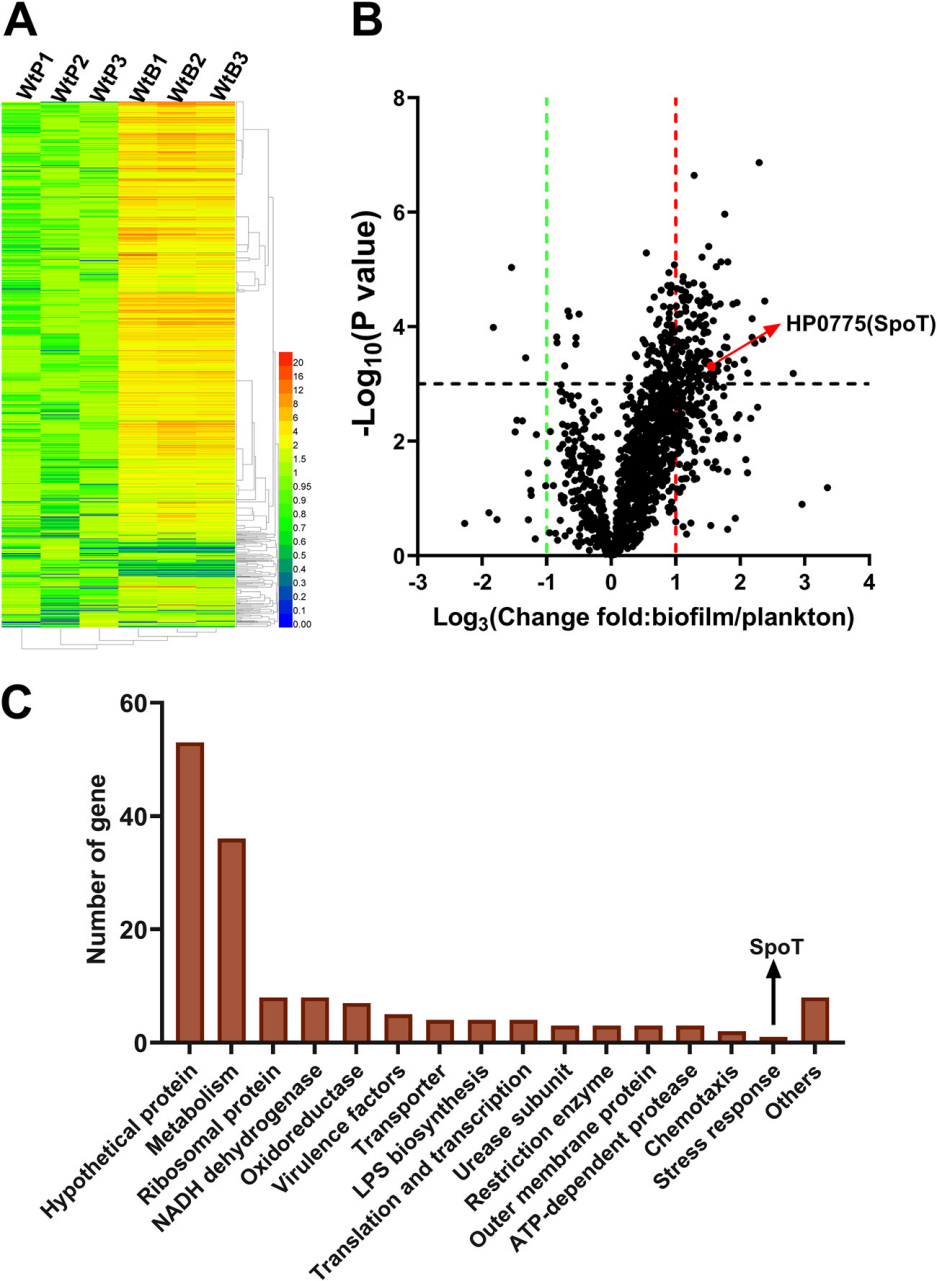
Analysis of differentially expressed genes in biofilms and planktonic cells of the wild-type Helicobacter pylori strain. (A) Heat map of transcripts expressed in biofilms (*n* = 3) and planktonic cells (*n* = 3). (B) Volcano plot analysis of expressed transcripts to identify the differentially expressed genes. (C) Functional classification of differentially expressed genes according to the KEGG GENES database. The number of annotated genes (*y* axis) is plotted against the KEGG categories (*x* axis). LPS, lipopolysaccharide.

To analyze the mechanism underlying H_2_O_2_-induced H. pylori biofilm formation, the transcriptomes of the planktonic WT strain (WtP) and the biofilm-forming WT strain (WtB) were comparatively analyzed. The transcriptome data were represented as a heat map (Fig. 2A).

The genes that were differentially expressed in the WtB and WtP strains were analyzed using a volcano plot (Fig. 2B). In total, 152 differentially expressed genes were identified. Analysis with the KEGG GENES database revealed that the differentially expressed genes can be classified into various functional components, including metabolism and enzymes (25), ribosomal proteins (8), oxidoreductases (7), regulatory genes (1), and transporters (efflux pumps) (Fig. 2C).

The stress response gene *spoT* (HP0775) is shown on the volcano plot (Fig. 2B). *spoT* encodes (p)ppGpp synthase/hydrolase (26). (p)ppGpp, which was first discovered in Escherichia coli by Michael Cashel in the 1960s (27), is involved in the regulation of the bacterial stringent/stress response. The stress response is an adaptive regulatory response of bacteria to stressful environments, such as nutritional deficiency, heat stress, and antibiotics (26).

The formation of biofilms is a bacterial stress response (28). Hence, we hypothesized that SpoT may play an important role in the oxidative stress-induced formation of H. pylori biofilms.

### SpoT promotes oxidative stress-induced H. pylori biofilm formation and confers multidrug resistance.

**FIG 3:**
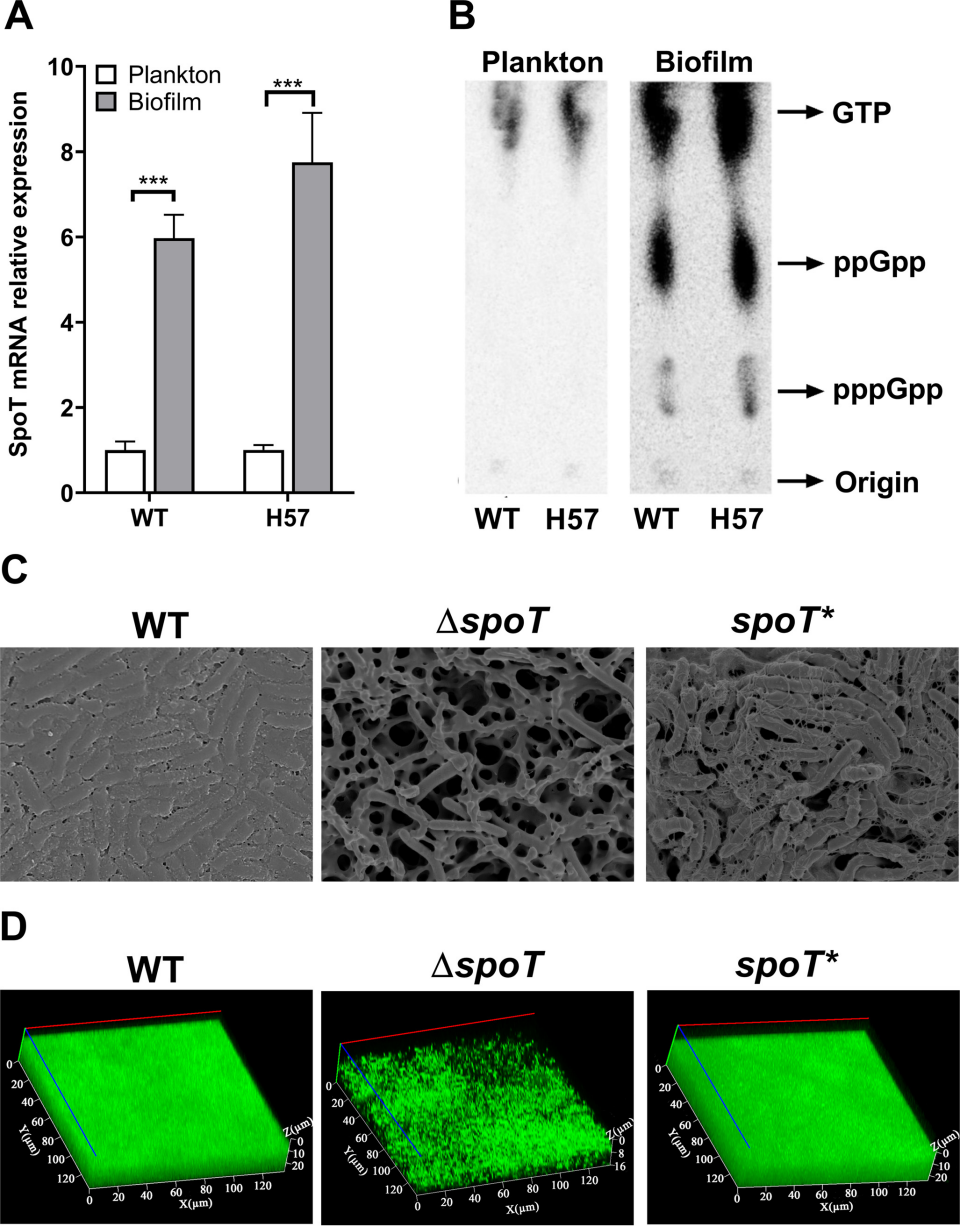
SpoT is involved in the H_2_O_2_-induced formation of biofilms by the Helicobacter pylori wild-type (WT) and H57 strains. (A) Levels of *spoT* mRNA expression in biofilm-forming and planktonic cells were examined using quantitative real-time PCR. The expression levels of target genes were normalized to those of 16S rRNA genes. Data are presented as means ± standard errors of the means from three independent experiments. Asterisks indicate significance by an unpaired Student *t* test (***, *P* <0.001). (B) The expression of (p)ppGpp (guanosine 3′-diphosphate 5′-triphosphate and guanosine 3′,5′-bispyrophosphate) was upregulated in biofilm-forming cells of the WT and H57 strains but not in planktonic cells. ^32^P-labeled nucleotides of formic acid extracts of H. pylori were detected using thin-layer chromatography. Planktonic H. pylori bacteria were cultured to the exponential phase. (C) Scanning electron microscopy images of WT, Δ*spoT*, and spoT* biofilms. In this experiment, a mature biofilm grown on a nitrocellulose membrane for 3 days was used. The planktonic bacteria were from the early-exponential-phase culture (OD_600_, 0.4 to 0.5). (D) Confocal laser scanning microscopy images of WT, Δ*spoT*, and spoT* biofilms. Cells stained with membrane-permeant SYTO 9 (green) and membrane-impermeant propidium iodide (red) were visualized using confocal microscopy.

The levels of SpoT expression in H_2_O_2_-induced biofilms of the WT and H57 strains were examined using quantitative real-time PCR (qRT-PCR). The results showed that *spoT* was upregulated in biofilm-forming cells from both the WT and H57 strains relative to expression in planktonic cells (Fig. 3A).

To further verify the level of SpoT expression in biofilms, the levels of ppGpp and pppGpp expression in planktonic and biofilm-forming cells of the WT and H57 strains were examined using a ^32^P-postlabeling/thin-layer chromatography (^32^P-TLC) assay (Fig. 3B). Relative to those in planktonic cells, the expression levels of ppGpp and pppGpp were significantly upregulated in biofilm-forming cells.

These results indicate that SpoT is involved in the oxidative stress-induced formation of H. pylori biofilms. Additionally, a *spoT* knockout strain (Δ*spoT*) and a complemented strain (spoT*) were constructed in order to comparatively analyze the biofilm-forming abilities of the Δ*spoT*, spoT*, and WT strains (Fig. 3C and D). As shown in Fig. 3C and D, the Δ*spoT* strain could not form a complete biofilm. Moreover, the biofilm-forming ability of the spoT* strain was similar to that of the WT strain.

Next, the susceptibilities of WT, Δ*spoT*, and spoT* biofilms to multiple antibiotics were comparatively analyzed (Table S1). The MIC value of penicillin G against the Δ*spoT* strain was 30-fold lower than that against the WT strain. The MIC value of tetracycline hydrochloride against the WT strain was 8-fold lower than that against the Δ*spoT* strain. Additionally, the MIC values of various antibiotics for the spoT* and WT strains were similar.

### WGCNA modules associated with H. pylori biofilm formation and SpoT.

**FIG 4:**
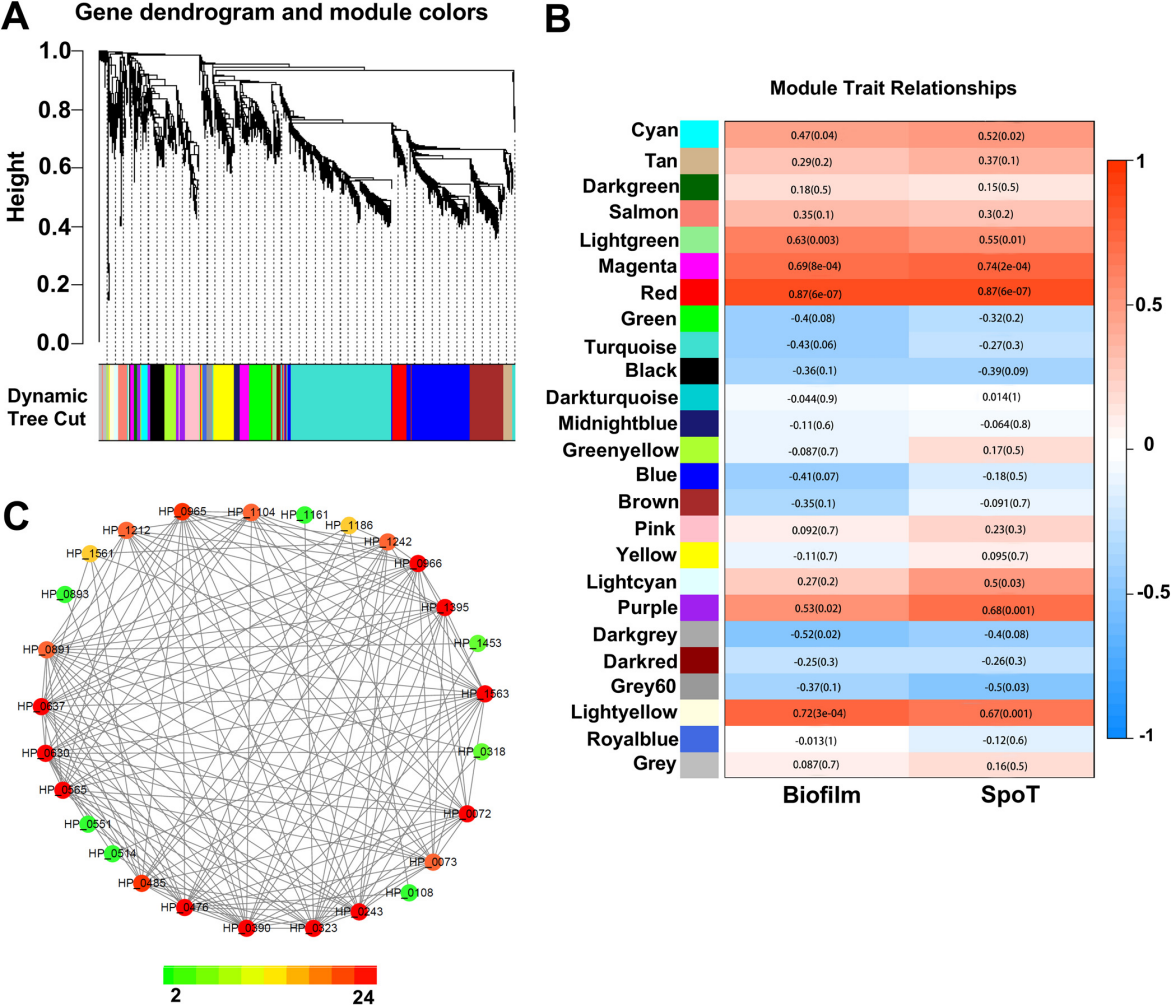
Weighted gene coexpression network analysis of genes that are differentially expressed in the biofilm-forming and planktonic cells of the wild-type (WT) and Δ*spoT* strains. (A) (Top) Hierarchical cluster tree showing 25 modules of coexpressed genes. Each differentially expressed gene is represented as a leaf on the tree, while each of the 25 modules is represented as a tree branch. (Bottom) Modules shown in colors (such as tan, red, and yellow) designated in panel B. (B) (Left) The 25 modules. (Center) Correlation between modules and SpoT/biofilm weight (with the corresponding *P* values shown in parentheses). (Right) Color scale showing module-trait correlation from −1 (blue) to 1 (red). (C) Cytoscape representation of coexpressed genes with edge weights of ≥0.10 in the “red” module. The number of edges of the genes ranges from 4 to 24 (color-coded from green through red according to the scale on the bottom). Member gene identifications are shown.

The biofilm-forming cells and planktonic cells of the Δ*spoT* strain (Δ*spoT*B and Δ*spoT*P cells, respectively) were also subjected to transcriptome sequencing. The transcriptome data of these cells were compared with those of the WT strain. Weighted gene coexpression network analysis (WGCNA) of 1,526 genes (Fig. S1) revealed 25 modules (Fig. 4A and B). Correlation analysis revealed that the “red” module, containing 62 genes, was correlated with *spoT* (*r* = 0.87; *P = *9.0 × 10^−7^) and biofilm formation (*r* = 0.87; *P = *9.0 × 10^−7^) (Fig. 4B). Therefore, these 62 SpoT-regulated genes in the “red” module were considered to play an important role in the oxidative stress-induced formation of H. pylori biofilms.

Cytoscape network visualization of 27 genes with WGCNA edge weights of >0.10 indicated that these genes are highly correlated. Of the 27 genes, 23 had five or more edges, while only 4 genes (*hp0893*, *hp1453*, *hp0318*, and *hp0108*) had low edge numbers (Fig. 4C).

Additionally, the “magenta” module, with 53 genes, and the “light yellow” module, with 43 genes, were correlated with H. pylori biofilm formation and SpoT. However, the correlations of the “magenta” and “light yellow” modules with biofilm formation and SpoT were lower than that of the “red” module.

According to the functional annotation of genes in the KEGG GENES database, 27 genes with WGCNA edge weights of >0.10 in the “red” module can be divided into eight categories, including outer membrane proteins, ABC transporters, oxidoreductases, and hydrolases (Table 1).

**TABLE 1:** Genes that may be involved in H. pylori biofilm formation and regulated by SpoT, according to WGCNA analysis^*a*^

Classification according to function	Predicted function	Locus tag
Outer membrane protein	Outer membrane protein (*omp30*)	HP_1395
ABC transporter	Iron(III) ABC transporter, periplasmic iron-binding protein (*ceuE*)	HP_1561
Oxidoreductase	DNA protection during starvation protein	HP_0243
	Cinnamyl-alcohol dehydrogenase ELI3-2 (*cad*)	HP_1104
	Flavodoxin (*fldA*)	HP_1161
	Catalase-like protein	HP_0485
	Alkyl hydroperoxide reductase (*tsaA*)	HP_1563
	Adhesin-thiol peroxidase (*tagD*)	HP_0390
	NADPH quinone reductase, modulator of drug activity (*mda66*)	HP_0630
Hydrolase	Urease subunit beta	HP_0072
	Urease subunit alpha	HP_0073
Genetic information processing	Glutamyl-tRNA synthetase (*gltX1*)	HP_0476
Metabolism	F_o_F_1_ ATP synthase subunit C (*atpE*)	HP_1212
Ribosomal protein	50S ribosomal protein L9	HP_0514
	50S ribosomal protein L31	HP_0551
Hypothetical protein	Hypothetical protein	HP_0108
	Hypothetical protein	HP_0318
	Hypothetical protein	HP_0565
	Hypothetical protein	HP_0637
	Hypothetical protein	HP_0891
	Hypothetical protein	HP_0893
	Hypothetical protein	HP_0965
	Hypothetical protein	HP_0966
	Hypothetical protein	HP_1453

a See Fig. 4C.

### Screening the key target genes involved in SpoT-regulated biofilm formation.

To further identify the key target genes involved in SpoT-regulated biofilm formation, the expression levels of nine key genes in the WT and H57 strains were analyzed using qRT-PCR (Table 2). These genes were functionally classified as outer membrane proteins, ABC transporters, and oxidoreductases, which may be involved in biofilm formation.

**TABLE 2:** qRT-PCR analysis of the relative gene expression difference between biofilm-forming and planktonic cells in the WT and H57 strains

Locus tag	Predicted function	BF/PKC fold change^*a*^ in the following strain:	
		WT	H57
HP_0243	DNA protection during starvation protein (*napA*)	4.62 ± 0.41***	7.52 ± 0.26***
HP_1104	Cinnamyl-alcohol dehydrogenase ELI3-2 (*cad*)	1.17 ± 0.10	5.60 ± 0.16**
HP_1161	Flavodoxin (*fldA*)	4.35 ± 0.38***	2.98 ± 0.32**
HP_0485	Catalase-like protein	2.00 ± 0.06***	3.27 ± 0.30***
HP_1563	Alkyl hydroperoxide reductase (*tsaA*)	1.59 ± 0.12**	2.44 ± 0.92
HP_0390	Adhesin-thiol peroxidase (*tagD*)	1.68 ± 0.08	4.54 ± 0.54***
HP_0630	Modulator of drug activity (*mda66)*	2.74 ± 0.27***	4.98 ± 0.46**
HP_1395	Outer membrane protein (*omp30*)	0.59 ± 0.02**	4.60 ± 0.11***
HP_1561	Iron(III) ABC transporter, periplasmic iron-binding protein (*ceuE*)	1.18 ± 0.08	2.13 ± 0.31**

a BF, biofilm-forming cells; PKC, planktonic cells. *P* values are indicated by asterisks as follows: **, *P* < 0.01; ***, *P* < 0.001.

As shown in Table 2, for the WT strain, the expression levels of two genes (*hp0243* [*napA*]) and *hp1161*) in biofilm-forming cells were upregulated by >4-fold over those in planktonic cells. In addition, for the H57 strain, the expression levels of the nine key genes in biofilm-forming cells were upregulated over those in planktonic cells, and the expression of *napA* was significantly upregulated, by >7-fold (Table 2). The fact that *napA* expression in biofilm-forming cells of both the WT and H57 strains was upregulated by >4-fold over that in planktonic cells indicates that *napA* may be involved in biofilm formation under oxidative stress conditions.

### SpoT regulates *napA* expression.

**FIG 5:**
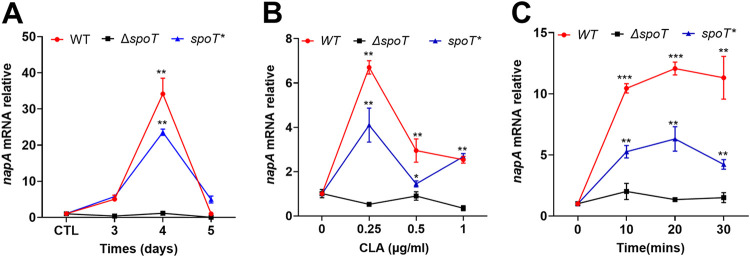
*napA* mRNA expression levels in the wild-type (WT), Δ*spoT*, and spoT* strains. (A) Expression of *napA* in the biofilms of the WT, Δ*spoT*, and spoT* strains for different durations. The planktonic cells served as a control. (B and C) Expression levels of *napA* in the WT, Δ*spoT*, and spoT* strains treated with different concentrations of clarithromycin (CLA) for 30 min (B) or with 0.25 μg/ml CLA for various durations (C).

The role of *spoT* in regulating *napA* expression was examined by comparatively analyzing the expression levels of *napA* in biofilm-forming cells of the WT, Δ*spoT*, and spoT* strains (Fig. 5A). *napA* expression was upregulated in the WT and spoT* strains on the third, fourth, and fifth days of biofilm formation. Additionally, *napA* expression was highest on the fourth day of biofilm formation in these strains but was not detected in the Δ*spoT* strain (Fig. 5A).

Previously, we had demonstrated that clarithromycin (CLA) could promote *spoT* expression (29). To examine the regulation of *spoT* expression by *napA*, the WT, Δ*spoT*, and spoT* strains were treated with different concentrations of CLA (0.25, 0.5, and 1 μg/ml) for 30 min. *napA* expression levels were examined using qRT-PCR. Treatment with 0.25 μg/ml of CLA was considered an optimal condition to induce *napA* expression in the WT and spoT* strains but not in the Δ*spoT* strain (Fig. 5B).

Next, *napA* expression levels in the WT, Δ*spoT*, and spoT* strains treated with 0.25 μg/ml CLA for 10, 20, or 30 min were examined using qRT-PCR. CLA time-dependently upregulated *napA* expression in the WT and spoT* strains. In contrast, CLA did not upregulate *napA* expression in the *ΔspoT* strain (Fig. 5C).

### NapA promotes H. pylori biofilm formation and confers multidrug resistance.

**FIG 6:**
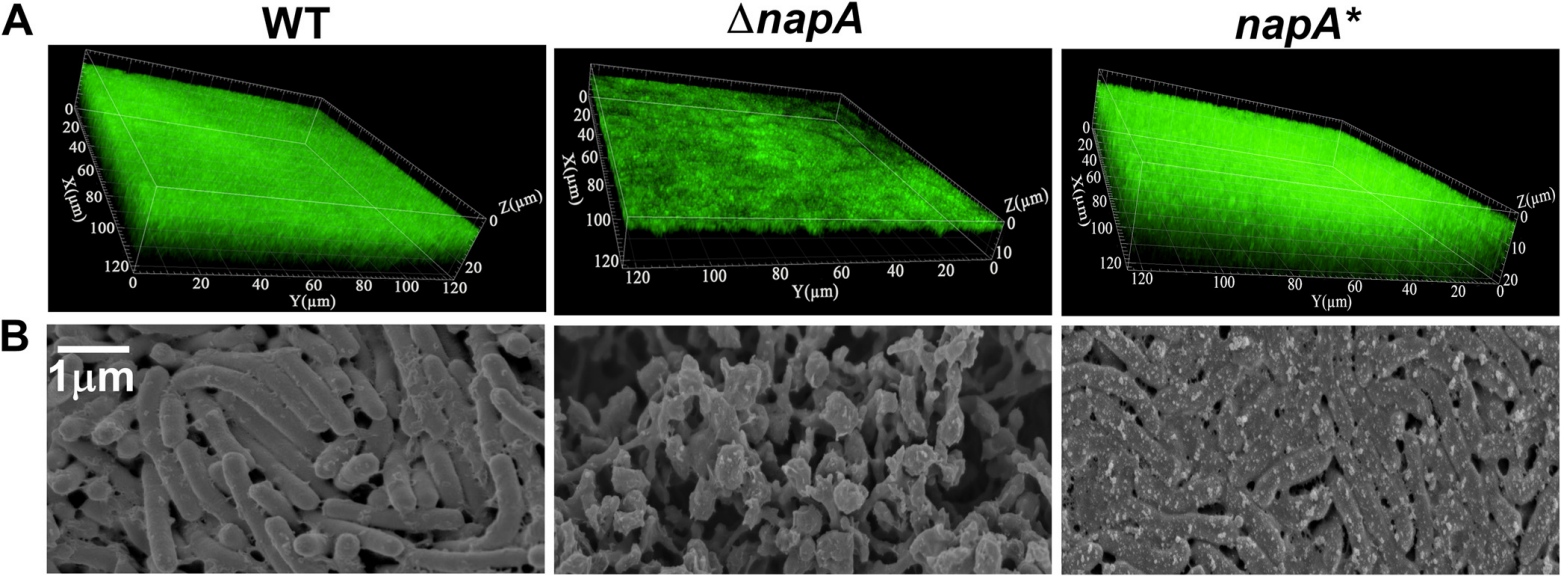
NapA is involved in the H_2_O_2_-induced formation of Helicobacter pylori biofilms. (A) Confocal laser scanning microscopy images of wild-type (WT), Δ*napA*, and napA* biofilms. Cells stained with membrane-permeant SYTO 9 (green) and membrane-impermeant propidium iodide (red) were visualized using confocal microscopy. (B) Scanning electron microscopy images of WT, Δ*napA*, and napA* biofilms. In this experiment, a mature biofilm grown on a nitrocellulose membrane for 3 days was used. The planktonic bacteria were from the early-exponential-phase culture (OD_600_, 0.4 to 0.5).

Previous studies have demonstrated that *napA* can protect H. pylori against oxidative stress (30), suggesting that *napA* may be involved in the oxidative stress-induced formation of H. pylori biofilms. The formation of biofilms in the WT, *napA* knockout (Δ*napA*), and *napA* complementation (napA*) strains was analyzed using SEM. Compared with the WT and napA* biofilms, the Δ*napA* biofilm exhibited loose bacterial arrangement, incomplete extracellular matrix formation, a higher number of cavities, and a spherical shape. The results of confocal laser scanning microscopy (CLSM) analysis and a LIVE/DEAD cell viability assay revealed that the Δ*napA* strain formed a thin biofilm (Fig. 6).

Next, we compared the growth curves of the WT, Δ*napA*, and napA* strains and found that *napA* knockdown did not affect the growth of H. pylori (Fig. S2). Analysis of the MIC values of various antibiotics revealed that the Δ*napA* strain was more sensitive to antibiotics, such as amoxicillin, clarithromycin, and tetracycline, than the WT and napA* strains (Table 3).

**TABLE 3:** MICs determined for the WT, Δ*napA*, and napA* strains in biofilm-forming and planktonic cells

Drug	MIC (μg/ml) for the following type of cells:					
	Planktonic			Biofilm forming		
	WT	Δ*napA*	napA*	WT	Δ*napA*	napA*
Amoxicillin	0.0625	0.0156	0.03125	5	2	5
Clarithromycin	0.0625	0.0078	0.0156	6.25	1	4
Penicillin G	0.0625	0.0156	0.0625	8	1	8
Tetracycline	0.125	0.03125	0.0625	10	2	8
Metronidazole	0.5	0.125	0.25	16	1	12

**FIG 7:**
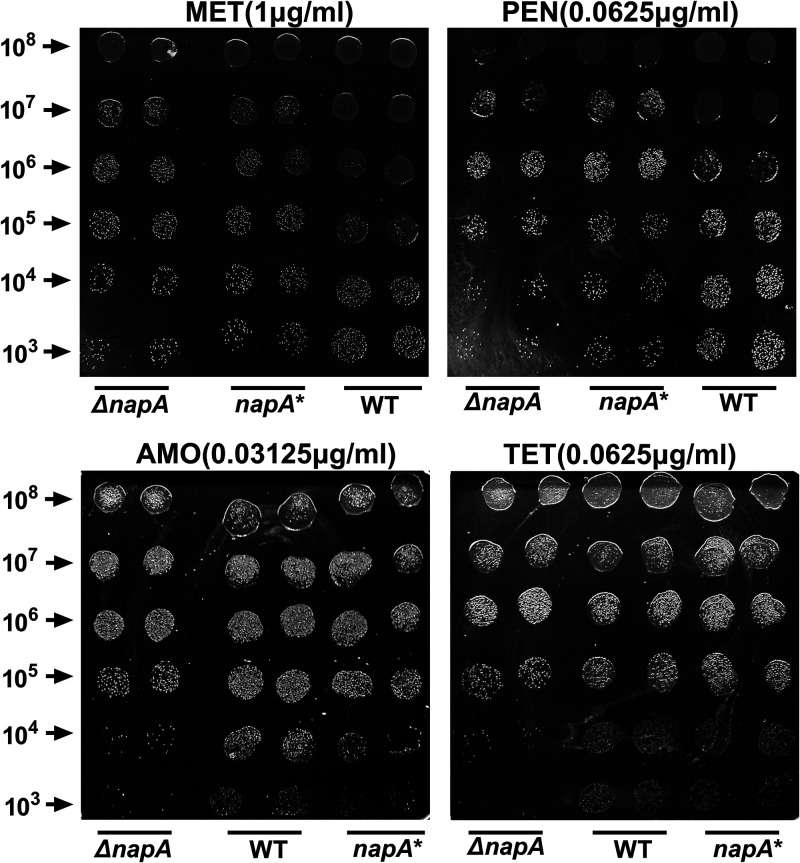
Effects of different antibiotics on the colony-forming abilities of the wild-type, Δ*napA*, and napA* strains. Serially diluted bacterial cultures were spotted onto Mueller-Hinton agar plates, and their colony-forming abilities in the presence of different antibiotics (metronidazole [MET], penicillin [PEN], amoxicillin [AMO], and tetracycline [TET]) were assessed after 3 days. The experiments were performed in triplicate, and representative examples are shown.

The viabilities of the three strains on plates supplemented with different antibiotics were comparatively analyzed. The number of clones formed by the Δ*napA* strain was significantly lower than the numbers formed by the WT and napA* strains (Fig. 7).

### *napA* knockout promotes oxidative stress-induced H. pylori genomic DNA damage.

**FIG 8:**
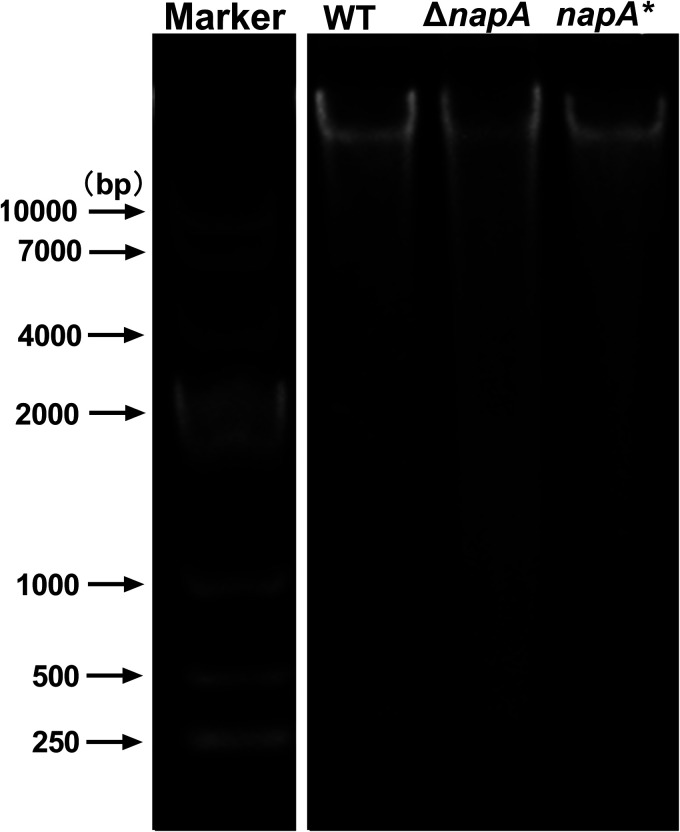
Agarose gel electrophoretic analysis of genomic DNA fragmentation in wild-type, Δ*napA*, and napA* cells. The sizes of DNA standards are shown on the left. The experiments were repeated three times and yielded similar results.

Previous studies have reported that *napA* can protect H. pylori against oxidative stress-induced genomic DNA damage. (31). Hence, DNA damage and fragmentation in the WT, Δ*napA*, and napA* biofilms were examined using electrophoresis (Fig. 8). The DNA fragmentation level was low in the WT strain, with 4 kb as the smallest fragment size. In contrast, the Δ*napA* strain exhibited significantly high levels of DNA fragmentation, and the size of the smallest fragment was approximately 1 kb. This indicated that relative to the WT strain, the Δ*napA* strain exhibited enhanced oxidative stress-induced genomic DNA damage.

### Vc and NAC exhibit anti-H. pylori biofilm activity and downregulate *napA* expression.

**FIG 9:**
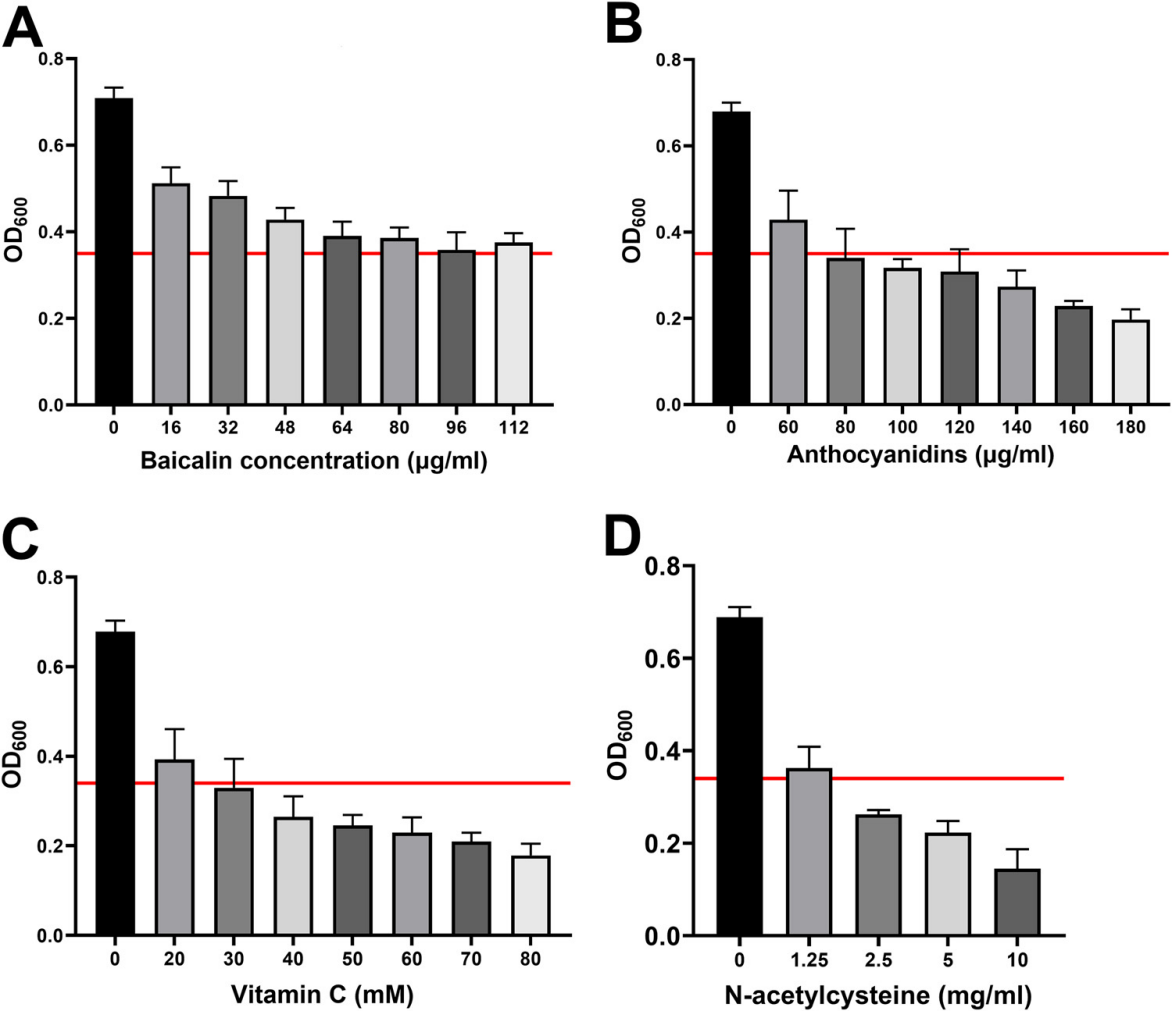
Effects of different antioxidants on biofilms of the wild-type strain. Biofilms grown for 3 days were treated with different concentrations of antioxidants for 24 h. The horizontal red line in each graph indicates a 50% reduction in the biomass of biofilms treated with antioxidants.

Next, we hypothesized that antioxidants may exhibit anti-H. pylori biofilm activity. In this study, the effects of various concentrations of antioxidants (32), such as baicalin, anthocyanins, vitamin C (Vc), and *N*-acetylcysteine (NAC), on H. pylori biofilms were examined. Baicalin exhibited weak anti-H. pylori biofilm activity. At a concentration of 112 μg/ml, baicalin could not decrease the biofilm mass by 50% (Fig. 9A). However, anthocyanins decreased the biofilm mass by >50% at treatment concentrations higher than 80 μg/ml (Fig. 9B).

**FIG 10:**
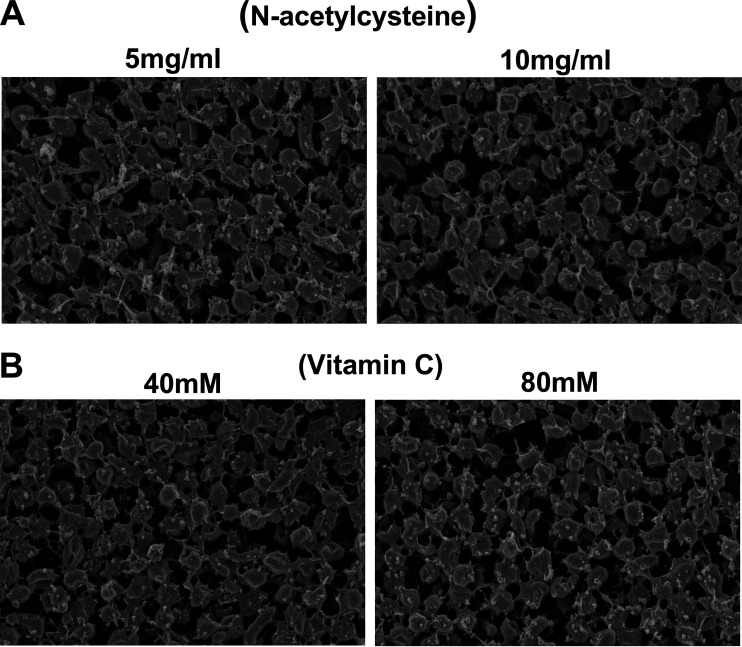
Scanning electron microscopy images of wild-type biofilms treated with different concentrations of *N*-acetylcysteine (A) or vitamin C (B) for 24 h.

Vc exhibited potent antibiofilm activity. At concentrations higher than 30 mM, Vc decreased the biofilm mass by >50% (Fig. 9C). NAC exhibited the strongest anti-H. pylori biofilm activity. Treatment with 2.5 mg/ml NAC decreased the biofilm mass by >50% (Fig. 9D). SEM analysis revealed that treatment with 5 mg/ml NAC resulted in almost complete loss of the extracellular matrix of the H. pylori biofilm (Fig. 10A). Similarly, treatment with 40 mM Vc resulted in the loss of the H. pylori biofilm extracellular matrix (Fig. 10B).

**FIG 11:**
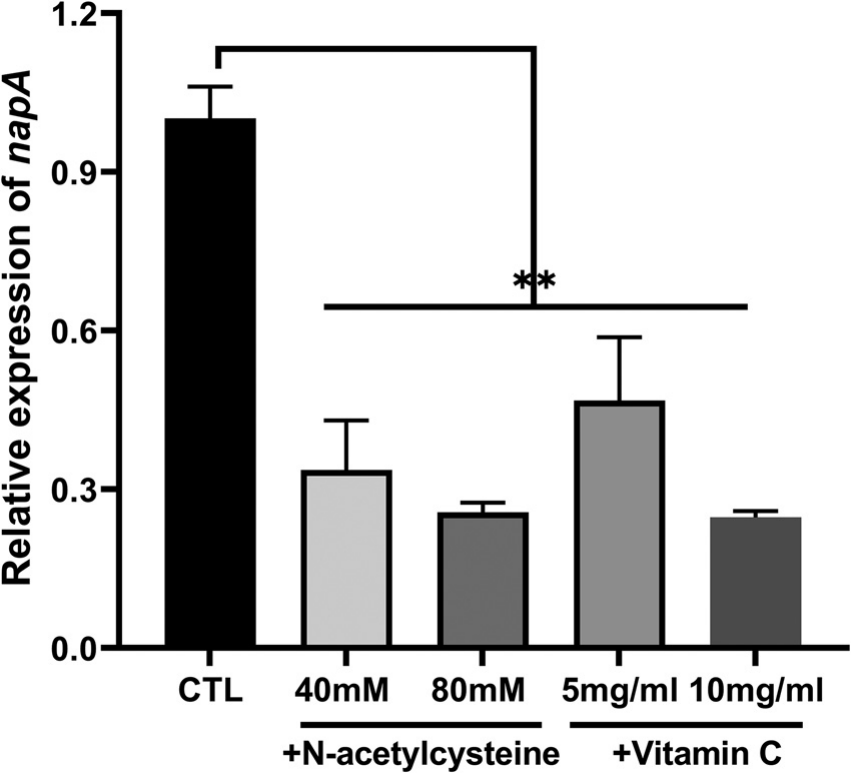
*napA* mRNA expression levels in the wild-type strain treated with different concentrations of vitamin C or *N*-acetylcysteine for 30 min. CTL, control.

This study demonstrated that NapA regulates the oxidative stress-induced formation of H. pylori. Hence, *napA* mRNA expression levels in the Vc-treated and NAC-treated groups were examined. Vc and NAC downregulated the expression of *napA* (Fig. 11).

## DISCUSSION

The incidence of drug resistance in H. pylori has increased in recent years. The formation of biofilms *in vivo* contributes to the development of multidrug resistance in H. pylori (5). Previous studies have demonstrated that a low concentration of ROS can activate the stress response mechanism of bacteria and consequently promote biofilm formation (33). In this study, H_2_O_2_-induced oxidative stress promoted the formation of H. pylori biofilms. SpoT positively regulated the expression of NapA and the oxidative stress-induced formation of H. pylori biofilms. Furthermore, *N*-acetylcysteine and Vc exhibited anti-H. pylori biofilm activity and downregulated *napA* expression.

Stress conditions, such as subinhibitory concentrations of antibiotics (34), amino acid starvation (35), and oxidative stress (28), promote the formation of biofilms in bacteria. For example, enhanced levels of Fe^3+^ can promote the production of ROS in the host and the formation of biofilms in Pseudomonas aeruginosa (36). Free radicals and ROS in cigarette smoke promoted the formation of Staphylococcus aureus biofilms (25). Endogenous H_2_O_2_ can stimulate the formation of Acinetobacter baumannii biofilms (33).

In this study, H_2_O_2_ did not affect the growth of planktonic bacteria at concentrations lower than 50 μM. This indicated that a low concentration of H_2_O_2_ does not affect the viability of H. pylori and cannot promote the formation of biofilms. At concentrations higher than 200 μM, H_2_O_2_ markedly inhibited the growth of H. pylori and the formation of biofilms. In this study, the optimal concentration range of H_2_O_2_ for inducing biofilm formation in H. pylori was determined to be 50 to 200 μM.

In-Ae Jang et al. demonstrated that treatment with 100 μM H_2_O_2_ promoted the formation of biofilms in Acinetobacter oleivorans DR1 (33). Excessive H_2_O_2_ (usually ≥50 μM) is cytotoxic to various animals, plants, and bacterial cell cultures (37). Therefore, this study treated bacterial cultures with H_2_O_2_ at a final concentration of 50 μM to simulate the physiological environment. Supplementation of the culture medium with 50 μM H_2_O_2_ promoted biofilm formation in the WT and H57 strains of H. pylori.

Various reductase systems of H. pylori are involved in scavenging ROS (18). Additionally, biofilm formation protects against ROS-mediated toxic effects on bacteria. Bacteria are enclosed within the extracellular matrix, which can inhibit ROS diffusion, after biofilm formation (19). In this study, H. pylori formed biofilms with a dense extracellular matrix upon stimulation with low concentrations of H_2_O_2_.

Genes involved in oxidative stress regulation in other bacteria, such as *oxyR*, *soxR*, *soxS*, *rpoS*, *lexA*, and *perR* (18), were not detected in the H. pylori genome. Some oxidative stress-regulatory genes, such as *oxyR* and *rpoS* (19), can also regulate the formation of bacterial biofilms. The genes that regulate the oxidative stress response in H. pylori include the cytoplasmic chemoreceptor (TlpD) (38, 39), iron uptake regulation (*fur*) (30), posttranscriptional regulation (*csrA*) (40), and stress regulation (*spoT*) genes (41).

In this study, SpoT expression was upregulated in oxidative stress-induced biofilms. In most bacteria, (p)ppGpp is synthesized by RelA and SpoT (42). However, analysis of the H. pylori genome revealed that it lacks the gene encoding RelA and contains only the gene encoding SpoT (43). This indicates that (p)ppGpp is synthesized by SpoT in H. pylori.

SpoT is reported to be involved in the adaptive response of H. pylori to various stress conditions, such as amino acid starvation, an acidic environment, or an oxidative stress environment (41, 44). Recently, we demonstrated that SpoT is involved in the regulation of H. pylori tolerance to multiple antibiotics and that biofilm formation is induced under nutrient starvation conditions (8). Additionally, recent studies have demonstrated that (p)ppGpp regulates the formation of biofilms in other bacteria, such as Enterococcus faecalis (45), Bordetella pertussis (46), and Vibrio cholerae (47). Inhibition of (p)ppGpp is reported to suppress bacterial biofilm formation (48).

In this study, SpoT expression was upregulated in H_2_O_2_-induced biofilm cells. The Δ*spoT* strain could not form a complete biofilm. This indicates that SpoT is involved in regulating the oxidative stress-induced formation of H. pylori biofilms.

NapA, which activates neutrophils, plays a major role in recruiting human neutrophils and monocytes to the infection site (49). Additionally, NapA stimulates the production of ROS in human neutrophils and monocytes (50). The sequence and structure of NapA are similar to those of Dps (DNA-binding protein from the starved cell) family proteins with iron-binding and DNA-protective activities (51). However, previous studies have demonstrated that the expression of NapA in H. pylori is not regulated by the iron content and that NapA is not involved in conferring metal resistance (52).

Proteins of the Dps family are expressed in most bacteria and accumulate under oxidative stress or nutrient starvation conditions. Some Dps family proteins protect the bacteria against oxidative stress (53).

The protective effect of NapA against oxidative stress was determined on the basis of upregulated NapA expression after the loss of the antioxidant enzyme AhpC (54). Upregulated NapA expression is the major pathway in H. pylori that compensates for the loss of major antioxidant stress factors (55). The survival rates of H. pylori *napA* mutants upon exposure to oxidative stress are lower than that of the WT strain, indicating that NapA protects H. pylori against oxidative stress damage (30). H. pylori NapA can bind DNA in the presence of iron ions. Therefore, the main role of NapA is to protect the DNA against iron-mediated oxidative stress damage (31). In this study, the Δ*napA* strain could hardly form a biofilm upon H_2_O_2_ stimulation but transformed into coccoid forms. Due to the lack of protection of the biofilm extracellular matrix, the genome of the Δ*napA* strain is more susceptible to H_2_O_2_ damage than the WT genome. In addition, there is evidence that the genomic DNA of the coccoid form of H. pylori is impaired by endogenous oxidative stress (56, 57).

Yang et al. have reported that NapA is involved in the formation of the extracellular matrix in H. pylori biofilms (58). The extracellular matrix of biofilms can prevent ROS diffusion (28). In this study, the biofilm formed by the Δ*napA* strain exhibited a thinner extracellular matrix and a higher number of cavities than those of the WT biofilm. Thus, ROS can penetrate the biofilm of the Δ*napA* strain.

An important mechanism by which (p)ppGpp induces global changes in transcription initiation involves the regulation of sigma factors. Under stringent environmental conditions, alternative sigma factors promote transcription, a process mediated by (p)ppGpp (42). In H. pylori, only two alternative σ factors (σ^54^ and σ^28^) have been reported (43). Previously, we had demonstrated that σ^54^ positively regulates NapA (59). Niehus et al. reported that the promoter sequence of σ^54^-dependent genes is TTTGCTT (60). Analysis of the upstream sequence of the putative ATG start codon in the *napA* open reading frame did not reveal a similar conservative sequence. However, analysis of the upstream sequence of the putative ATG start codon in the σ^28^ open reading frame revealed a potential conserved sequence (TTTGCTT), which may be recognized by σ^54^ (see Fig. S3A in the supplemental material). This indicates that σ^54^ can upregulate σ^28^. The expression levels of σ^28^ in the biofilm cells of the H. pylori WT and σ^54^ knockout strains were comparatively analyzed using qRT-PCR. σ^28^ expression was not upregulated in the σ^54^ knockout strain (Fig. S3B).

Josenhans et al. reported that the promoter sequence of genes regulated by σ^28^ is TAAAXXXXXXXXXXCCGAT (61). Analysis of the upstream sequence of the *napA* start codon revealed a similar conserved sequence (Fig. S4A). Therefore, σ^28^ may activate the transcription of *napA* by binding to its promoter. Comparative analysis of *napA* mRNA expression in the biofilm cells of the H. pylori WT and σ^28^ knockout strains revealed that *napA* expression was not upregulated in the σ^28^ knockout strain (Fig. S4B).

Further, σ^54^ expression levels in the biofilm cells of the WT, Δ*spoT*, and spoT* strains were examined. The analysis revealed that SpoT can upregulate σ^54^ expression (Fig. S5).

Thus, SpoT could upregulate *napA* transcription by activating σ^54^. Further, σ^54^ directly binds to the σ^28^ promoter to activate its transcription. σ^28^ subsequently promotes *napA* transcription by directly binding to the *napA* promoter and consequently facilitates the adaptation of H. pylori to the oxidative stress environment.

Previous studies have demonstrated that NAC can downregulate the production of the extracellular polysaccharide matrix and consequently inhibit the formation of biofilms in various bacteria (62). In this study, NAC inhibited the formation of H. pylori biofilms *in vitro* and exhibited antibiofilm activity. The administration of NAC before antibiotics can improve the clearance of drug-resistant H. pylori (11).

Various studies have demonstrated that Vc, a major dietary micronutrient, exerts growth-inhibitory effects against mycobacteria (63). Vc and NAC have been reported to synergistically potentiate the growth-inhibitory activity of antibiotics (64). Recent studies have reported that low concentrations of Vc exhibit antibiofilm activity in Bacillus subtilis by downregulating the synthesis of extracellular polymers (22). This may be attributed to the mucolytic activity of *N*-acetylcysteine or the structural homology between Vc and AI-2 (inhibition of AI-2-related quorum sensing) (5, 22). In this study, Vc and NAC exhibited similar anti-H. pylori biofilm activities. In addition to the reasons mentioned above, the downregulation of *napA* mRNA expression may be involved.

This study reported a novel mechanism of H. pylori biofilm formation under oxidative stress conditions. SpoT-regulated NapA promoted H_2_O_2_-induced biofilm formation in H. pylori.

## MATERIALS AND METHODS

### Bacterial strains, culture medium, and growth conditions.

H. pylori strain 26695 was s kind gift from Zhang Jianzhong, Chinese Center for Disease Control and Prevention. The strain was resuscitated on Mueller-Hinton agar (Oxoid, England) containing 5% sterile sheep blood and was cultured in a microaerobic environment (5% O_2_, 10% CO_2_, and 85% N_2_) at 37°C.

The liquid medium used to culture H. pylori was Brucella broth supplemented with 10% newborn calf serum. The bacteria were cultured at 37°C and 120 rpm in a shaker. The mutant strain and the complementation strain were cultured on agar plates containing 25 μg/ml kanamycin or 25 μg/ml chloramphenicol (both from Sigma-Aldrich, St. Louis, MO), respectively. These strains were stored in brain heart infusion medium (Oxoid, England) at −80°C with 20% (vol/vol) glycerol.

The clinical isolate H57 was isolated from a patient with a gastric ulcer at Qiannan People’s Hospital in Guizhou Province. The patient’s H. pylori infection was not alleviated even after three rounds of standard triple therapy. The patient signed an informed consent form before undergoing gastroscopy to isolate the bacteria. The study was approved by the Ethics Committee of the Shandong University School of Medicine (protocol number ECSBMSSDU2020-1-021).

The isolated H. pylori strains were identified on the basis of Gram staining and urease tests.

The TOP10 strain of E. coli (Tiangen, Beijing, China) was cultured in Luria-Bertani medium at 37°C.

### Biofilm construction.

Recent studies have demonstrated that low concentrations of oxidative stress-inducing agents can activate the bacterial stress response mechanism and stimulate the formation of biofilms (33, 65). H_2_O_2_ was added (final concentration, 50 μM) to Brinell agar medium (at 50°C), which was then poured onto a petri plate. The bacteria were allowed to form a biofilm by the “colony biofilm” method (66).

A sterilized and dried nitrocellulose filter (NC) membrane (1 cm^2^) was placed on fresh Brinell solid medium. The log-phase culture of H. pylori was resuspended in Brinell liquid medium to adjust the initial optical density at 600 nm (OD_600_) to 0.2. Next, 25 μl of the bacterial suspension was inoculated onto the NC membrane and allowed to dry partially. The plates were incubated upside down in a three-gas constant-temperature incubator. The bacteria were cultured for 4 days at 37°C in a microaerobic environment (85% N_2_, 10% CO_2_, and 5% O_2_).

### Growth curve of the biofilm.

The biofilm attached to the NC membrane was removed every 12 h and was washed repeatedly with sterile phosphate-buffered saline (PBS; pH 7) to obtain the planktonic bacteria attached to the surface of the biofilm. The cells in the biofilm were scraped and resuspended in 1 ml of liquid medium. The absorbance (expressed as the OD_600_) of the suspension was measured. The experiment was repeated three times. The growth curve of the H. pylori biofilm was generated based on the absorbance values.

### SEM.

The samples were subjected to SEM by following the standard procedure. The biofilm was gently washed three times with autoclaved PBS to remove planktonic bacteria from the surface. Next, the bacterial cells were fixed in 2.5% glutaraldehyde for 2 h at 4°C, washed three times with coconut oleate buffer, and dehydrated in an ethanol series (25%, 50%, 75%, 95%, and 100%). The sample was freeze-dried, sputtered, gilded, and observed under a scanning electron microscope.

### CLSM.

The thickness of the biofilm was analyzed using CLSM. The mature biofilm was washed multiple times with PBS to remove the planktonic bacteria attached to the surface. The biofilm was incubated with a fluorescent dye (LIVE/DEAD BacLight bacterial viability kit; Invitrogen, USA) for 20 min in the dark. Next, the sample was washed with PBS and placed on a glass slide. The sample was then incubated with an appropriate amount of antiquench agent, sealed with gum, and observed under a confocal laser scanning microscope.

### TLC.

To determine the levels of (p)ppGpp in the planktonic and biofilm-forming bacteria, (p)ppGpp was labeled with ^32^P. The planktonic and biofilm-forming cells were washed with PBS, and their OD_600_ values were adjusted to 0.2. The bacterial cells were centrifuged, and the supernatant was discarded. The cells were incubated with KH_2_PO_4_ and were labeled with 100 μCi/ml ^32^P at 37°C and 120 rpm for 3 h. Next, the samples were centrifuged, rinsed, resuspended in 50 μl of KH_2_PO_4_, incubated with an equal volume of 2 M formic acid, frozen at a low temperature, thawed at room temperature, and centrifuged at 13,000 rpm for 5 min after several freeze-thaw cycles. The supernatant (2.5 μl) was spotted onto polyethyleneimine cellulose-coated plates. The sample was dried and subjected to chromatography with 1.5 M KH_2_PO_4_ for 1 to 2 h. The chromatographic plate was dried in a fume hood. The phosphor screen was developed overnight and scanned.

### Evaluation of antibiotic sensitivity.

The sensitivities of H. pylori to various antibiotics were evaluated using the agar dilution method reported by Osato et al. (67). The following six antibiotics were used to evaluate the antibiotic sensitivities of different strains: penicillin G, amoxicillin, clarithromycin, tetracycline hydrochloride, ciprofloxacin, and metronidazole.

The planktonic bacterial culture (OD_600_, 0.8) was plated onto agar plates containing 2-fold serial dilutions of antibiotics. All plates were incubated at 37°C for 48 h under microaerobic conditions. The MIC and minimal bactericidal concentration (MBC) values were determined.

To evaluate the antibiotic sensitivities of biofilm-forming bacteria, the mature biofilms attached to the NC membranes were incubated for 12 h in liquid media containing different concentrations of antibiotics. The bacterial cells were washed three times with PBS and were resuspended in the liquid medium. The bacterial suspension (OD_600_, 0.8) was inoculated onto agar plates without antibiotics and was incubated at 37°C for 48 h to determine the MIC and MBC values.

To determine the sensitivities of bacteria to different antibiotics, a colony-forming assay was performed. Bacteria were cultured in liquid culture medium (approximately 10^8^ CFU/ml). The bacterial suspension serially diluted in PBS (5 μl) was plated onto Mueller-Hinton agar medium containing 5% sterile sheep blood and inhibitory concentrations of the antibiotics to allow the formation of a lawn. The bacteria were cultured in a microaerobic environment (5% O_2_, 10% CO_2_, and 85% N_2_) at 37°C for 3 days. The CFU of different strains were recorded. All experiments were performed at least three times.

### Evaluation of the effects of antioxidants on H. pylori biofilms.

The effects of antioxidants (baicalin, Vc, anthocyanin, and NAC) on H. pylori biofilms were examined. Biofilms grown for 3 days were treated with different concentrations of antioxidants for 24 h. The biofilm without antioxidant treatment was used as a control. Next, the biofilms were washed three times with sterile water to remove loosely attached bacteria and were dried at room temperature for 30 min.

Samples for SEM analysis were prepared by a standard procedure. The biofilm was stained with 1% crystal violet for 5 min, washed five times with sterile water to remove excess dye, and dried at 25°C for 1 h. Next, the stain was solubilized using absolute ethanol (1 ml) for 15 min. The absorbance of the solution (200 μl) at 600 nm was measured.

### mRNA sequencing.

mRNA high-throughput sequencing service was provided by CloudSeq Biotech (Shanghai, China). The H. pylori WT and Δ*spoT* strains cultured to the logarithmic phase or those that had formed biofilms were collected and divided into the following four groups according to the physiological state of the bacteria: WtP, WtB, Δ*spoT*P, and Δ*spoT*B cells. The bacterial cells were collected three times independently in each group.

The rRNAs in total RNA (1 μg) were removed by using Ribo-Zero rRNA removal kits (Illumina, San Diego, CA, USA) according to the manufacturer's instructions. RNA libraries were constructed with rRNA-free RNA by using the TruSeq Stranded Total RNA library prep kit (Illumina, San Diego, CA, USA) according to the manufacturer’s instructions. The quality and quantity of the libraries were determined using the Bioanalyzer 2100 system (Agilent Technologies, Inc., USA). The libraries (10 pM) were denatured, captured on Illumina flow cells, amplified *in situ* as clusters, and subjected to 150-cycle sequencing on an Illumina HiSeq sequencer according to the manufacturer’s instructions. The quality of paired-end reads was determined using Q30. The 3′-end adaptor was trimmed, and low-quality reads were removed using Cutadapt software (v1.9.1). Next, the high-quality trimmed reads (clean reads) were aligned with the H. pylori 26695 reference genome (ASM852v1) using HISAT2 software (v2.0.4). Based on the Ensembl gene annotation file, the gene expression level (expressed as fragments per kilobase per million mapped reads [FPKM]) was analyzed using Cuffdiff software (part of Cufflinks). The fold change in expression level and the *P* value were calculated based on FPKM to identify the differentially expressed mRNAs.

### RNA extraction and qRT-PCR.

Total bacterial RNA was extracted using the TRIzol reagent (Invitrogen, Carlsbad, CA, USA) and was reverse transcribed into cDNA using the PrimeScript RT kit (TaKaRa, Otsu, Shiga, Japan). The reaction mixture comprised 2.5 μl diluted cDNA, 0.4 μl primer mixture, 5 μl TB green *Premix Ex Taq* (TaKaRa), and 2.1 μl double-distilled water. qRT-PCR analysis was performed using an ABI Prism 7500 sequence detection system (Applied Biosystems, Carlsbad, CA). The dissociation curve was analyzed to verify the homogeneity of the product. The analysis was repeated three times. The internal reference gene for qRT-PCR analysis was 16S rRNA. The expression levels of the target genes were analyzed using the ΔΔ*C_T_* method.

### Construction of *napA* and *spoT* knockout and complementation strains.

Previously, we had successfully constructed the Δ*spoT* and spoT* strains (29). The plasmids (pILL570 and pUC18K2) used to construct mutants were provided by Agnès Labigne (Pasteur Institute).

The method of construction of the Δ*napA* strain was identical to that of the Δ*spoT* strain. Briefly, *napA* in the H. pylori 26695 genome was inactivated by the insertion of *aphA-3* (encoding kanamycin).

The *napA* complementation (napA*) strain was constructed by inserting WT *napA* between *hp0203* and *hp0204*, which contain the untranslated regions of the H. pylori chromosome. Briefly, *napA* along with the promoter region was PCR amplified and cloned into the PstI and XhoI sites of pBHKP252 (provided by Bi Hongkai of Nanjing Medical University). The recombinant plasmid was introduced into the Δ*napA* strain through natural transformation, and the recombinant colonies were isolated on Columbia blood agar plates containing chloramphenicol (10 μg/ml). Finally, the successful construction of the napA* strain was verified using PCR and sequencing. The primers used in this experiment are shown in Table S2.

### WGCNA.

A WGCNA network (68) was generated using the following four sets of transcriptome data: WtP (*n* = 3), WtB (*n* = 3), Δ*spoT*P (*n* = 3), and Δ*spoT*B strain (*n* = 3). A consensus network along with module statistics was generated by following the method of Langfelder et al. (69). The similarity matrix was calculated from the H. pylori transcriptome data based on the Pearson correlation coefficient. The exponential function was used as the adjacency function to determine the optimal parameters of the adjacency function according to the size of the transformed adjacency matrix *R*^2^, which results in strong biological significance. The topological overlap metric (TOM) (70) is derived from the adjacency matrix. Cluster analysis was performed on the results of gene clustering. The height of the hierarchical cluster tree was adjusted so that the smallest module contained at least 20 genes. The correlation between the gene module and the sample clinical indication matrix was analyzed. The correlation between each clinical indication and each module was calculated. The module with the strongest correlation with the target clinical indication was selected. Furthermore, the module was considered significant when the *P* value was <0.05. The most significant module (color coded red in Fig. 4B) of 24 genes with WGCNA edge weights of >0.10 was represented using Cytoscape, v3.1 (71).

### Statistical analysis.

Data are presented as means ± standard errors of the means. Statistical significance was determined using the unpaired Student *t* test, and the *P* values were corrected by the Sidak-Bonferroni method for multiple comparisons. *P* values of <0.05 were considered statistically significant. The results were analyzed using GraphPad Prism software (GraphPad Software Inc., La Jolla, CA, USA).

### Data availability.

The data supporting the findings are presented in the article and have been deposited in the NCBI database (BioProject accession no. PRJNA648673 and BioSample accession no. SAMN15644285 to SAMN15644288). The other relevant files can be acquired from the authors upon request.

## Supplementary Material

Supplemental file 1
