# Supplementary material for: SpoT-Mediated NapA Upregulation Promotes Oxidative Stress-Induced Helicobacter pylori Biofilm Formation and Confers Multidrug Resistance
Source: Antimicrob Agents Chemother. 2021 Apr 19;65(5):e00152-21. doi: 10.1128/AAC.00152-21 (PMC8092859; doi:10.1128/AAC.00152-21)
Supplement: Supplemental file 1 [file AAC.00152-21-s0001.pdf]

Supplemental Material FOR Publication

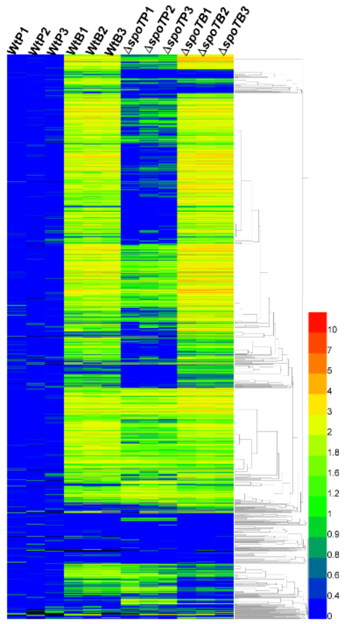

Fig. S1 Heatmap of transcripts expressed in the biofilms (n=3) and planktonic cells (n=3) of the wildtype (n=3) and Δ*spoT* strains.

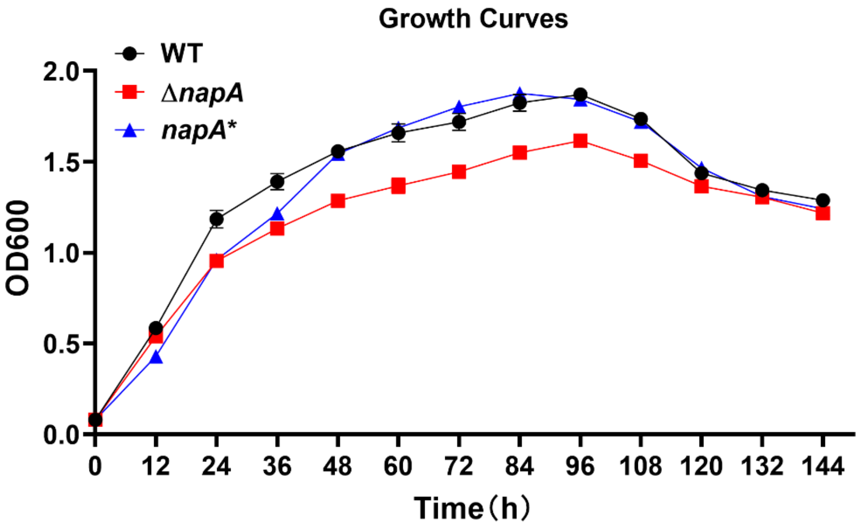

Fig S2 Growth curves of the wildtype (WT), Δ*napA* and *napA strains .**

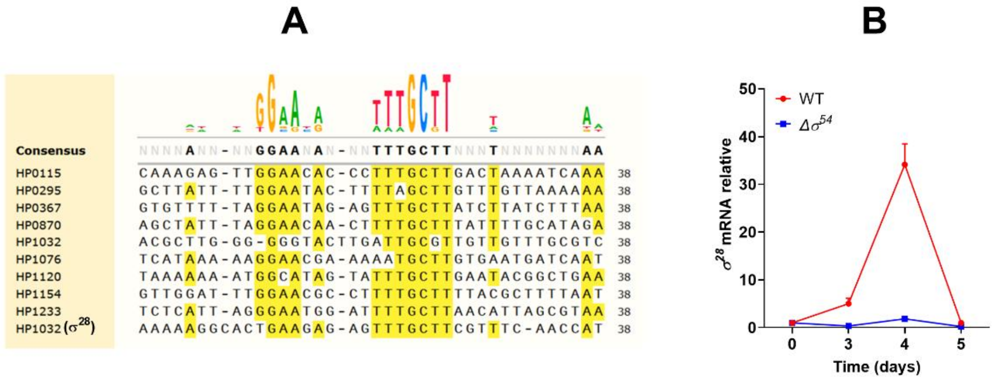

Fig. S3 σ^{54} may regulate σ^{28}. (A) Alignment of promoter sequences of nine known σ^{54}-dependent genes (Niehus et al., 2004) with those of Hp1032 (σ^{28}). The numbers to the right of the sequences indicate the distance from the putative ATG start codon of the open reading frames. The derived consensus sequence is shown in the bottom lane. The shading was performed using the Snapgene software (GSL Biotech; available at snapgene.com). Sequence logo is represented at the top and the height of the letter corresponds to the degree of conservation of the corresponding nucleotide. (B) The expression of σ^{28} in the biofilms of the wildtype and Δ*rpoN* ($\Delta\sigma^{54}$) strains at different durations. The planktonic cells served as a control.

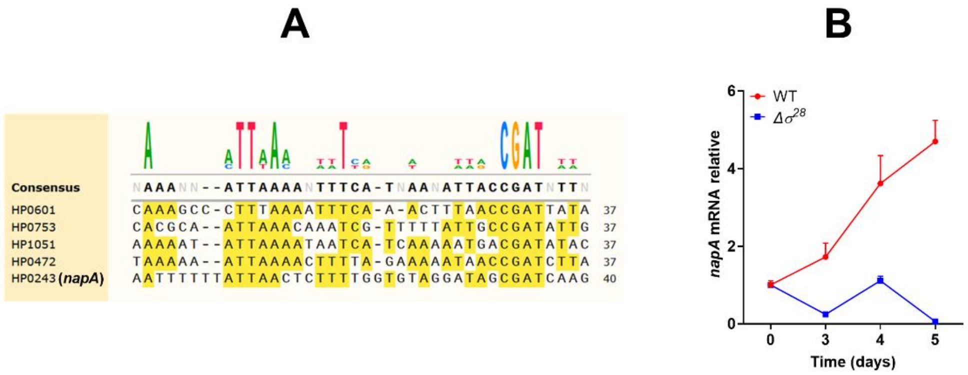

Fig. S4 σ^{28} may regulate *napA*. (A) Alignment of promoter sequences of four known σ*54*-dependent genes (Josenhans et al., 2002) with those of Hp0243 (*napA*). Numbers to the right of the sequences indicate the distance from the putative ATG start codon of the open reading frames. The derived consensus sequence is shown in the bottom lane. The shading was performed using the Snapgene software. Sequence logo is represented at the top and the height of the letter corresponds to the degree of conservation of the corresponding nucleotide. (B) The expression level of *napA* in the biofilms of wildtype and Δ*rpoN* ($\Delta\sigma^{28}$) strains at different durations. The planktonic cells served as a control.

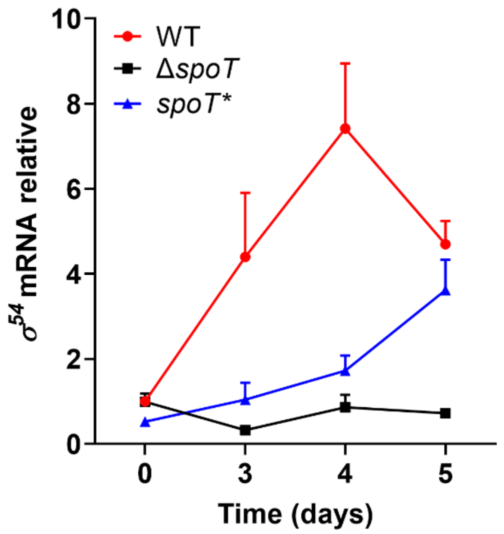

Fig. S5 Expression level of σ^{54} in the wildtype, Δ*spoT*, and *spoT biofilms at different durations. The planktonic cells served as a control.**

Table S1 MICs determined for WT, Δ*spoT*, *spoT** strains in biofilm-forming cells induced by nutrition deficiency or oxidative stress.

**Drug**	**MIC(μg/ml)**					
	Nutritional deficiency			Oxidative stress		
	WT	Δ*spoT*	*spoT**	WT	Δ*spoT*	*spoT**
**Penicillin G**	2.5	0.234	2.5	7.5	0.234	4.375
**Ciprofloxacin**	5	2	5	7.5	1.5	5
**Amoxicillin**	2.5	0.486	1.25	5	0.78	5.625
**Clarithromycin**	5	1.56	2.5	6.25	3.75	7.5
**Metronidazole**	5	3.125	5	15	5	10
**Tetracycline hydrochloride**	10	1.875	5	10	1.25	7.5

Table S2 Primers used in this study [a]

Name	Forward primers	Reverse primers
16S rRNA	GCTAAGAGATCAGCCTATGTCC	TGGCAATCAGCGTCAGGTAATG
hp0243	GAATGTGAAAGGCACCGATT	GGTGATGCCCTAATTGAACG
hp1563	GAAAAAGGCGGTATTGGTCA	GATCACCGCATGCCTTACTT
hp0630	TCTTTGGGGCTAGAAGTGGA	TTCACAATCCAAGGCTCTCC
hp0390	ATCGGTTTGTTTGCTCCAAG	AACAGCACGCCGTAATTTTC
hp1395	TTTGGCTTACGCCTTTATGG	TTAAAGGTTTTGGGATCAGC
hp1104	AGGAAAATGCACCTTGTTGG	ATCATTTGGCACAGCAATCA
hp1161	CAAACGAGCAAAAACAGCAA	CCTCAAATGATGGACGCTCT
hp0485	GGTGTGGGCCTTGTAAGATG	AAGCGTAGGAATGCTCCTGA
hp1561	TGCAAGAAAGCGTAGGGACT	GCGCTATCGTTCGCTAAATC
hp1032	GAATGCCCAAAGGAATTCAA	AGCGAGATCGTCTTGATGGT
hp0741	CAGCGGGTTGAATAATGAGG	ACTAAAACGCTTGCGCACTT
***hp0243*S**	AACTGCAGTAAAGGCATTCAAGCCAACC	CGGAATTCAATCGGTGCCTTTCACATTC
***hp0243*X**	CGGGATCCGCCAAGTTGCAAAAATCCAT	CCATCGATAAGGGGCATAAGGGCTAAAA
***hp0243*C**	CCGCTCGAGATGAAAACATTTGAAATTC	CGGGATCCTTAAGCCAAATGGGCTTGCA

[a] Underlining indicates nucleotides that were added at the 5’ end to create a restriction site.

Reference

Niehus E, Gressmann H, Ye F, Schlapbach R, Dehio M, Dehio C, Stack A, Meyer TF, Suerbaum S, Josenhans C. 2004. Genome-wide analysis of transcriptional hierarchy and feedback regulation in the flagellar system of Helicobacter pylori. Mol Microbiol 52:947–961.

Josenhans C, Niehus E, Amersbach S, Hörster A, Betz C, Drescher B, Hughes KT, Suerbaum S. 2002. Functional characterization of the antagonistic flagellar late regulators FliA and FlgM of Helicobacter pylori and their effects on the H. pylori transcriptome. Mol Microbiol 43:307–322.
